# Global burden of reported lower respiratory system fungal infection

**DOI:** 10.3389/fcimb.2025.1542922

**Published:** 2025-02-14

**Authors:** Zheng Wang, Mengshu Pan, Jie Zhu

**Affiliations:** ^1^ Department of Critical Care Medicine, Shandong Provincial Hospital Affiliated to Shandong First Medical University, Jinan, Shandong, China; ^2^ Primary Care Department, Second Affiliated Hospital of Anhui Medical University, Hefei, Anhui, China; ^3^ Department of Infectious Disease, Second Affiliated Hospital of Anhui Medical University, Hefei, Anhui, China

**Keywords:** global health, lower respiratory tract fungal infections, age-standardized disability-adjusted life-year rates, age-standardized mortality rates, join-point regression analysis

## Abstract

**Background:**

The epidemiological trend of lower respiratory tract fungal infections remains unclear. This study aims to quantify the global burden of these infections from 1990 to 2021 using data from the Global Burden of Disease Study 2021.

**Methods:**

Data were analyzed at global, regional, and national levels, considering factors such as age, gender, region, and socio-demographic index (SDI). Key indicators included age-standardized disability-adjusted life-year (ASDR) and age-standardized mortality rates (ASMR).

**Results:**

On a global scale, the burden of lower respiratory fungal infections decreased significantly from 1990 to 2021, with a decline in mortality rates. Regionally, substantial disparities were observed among the 21 geographic super-regions. Nationally, Argentina experienced the greatest increase in ASDR and ASMR, while Finland showed the largest decrease, with average annual percentage changes (AAPC) below -5 for both indicators. Sex-based analysis revealed a notably higher burden in males compared to females. ASDR and ASMR were negatively correlated with SDI in most regions.

**Conclusion:**

Although the global burden of lower respiratory tract fungal infections has decreased, it remains a substantial public health issue, particularly in low-SDI regions. There is an urgent need to implement targeted preventive measures to address this ongoing challenge.

## Introduction

Lower respiratory infections constitute a primary cause of illness and death worldwide (GBD 2016 Lower Respiratory Infections Collaborators, 2018). The causes of lower respiratory tract infections can be either viral, bacterial or fungal (Lukšić et al., 2013). Lower respiratory tract fungal infections have turned into a grave issue that should not be overlooked in the present world (Martins-Santana et al., 2023). Socioeconomic and geo-ecological traits, together with the escalating number of at-risk populations, serve as the principal determinants of variations in the incidence and prevalence of fungal disease worldwide (Bongomin et al., 2017). Along with the growth of the global population, the aggravation of population aging, the rise in the quantity of immunosuppressed individuals, and the progress of medical technology, the incidence and severity of fungal infections are continuously escalating. A study indicated that around 6.5 million cases of invasive fungal infections arise annually, along with 3.8 million fatalities. Of these, approximately 2.5 million deaths can be directly ascribed to it (Denning, 2024).

It should be noted that The epidemiological profile of fungal infections differs in various geographical areas, relying on multiple elements such as at-risk persons, socioeconomic characteristics, and the prevalence of fungi associated with geo-ecological traits, all of which exert considerable influences on health (Brown et al., 2012). In 2018, around 660,000 cases of fungal infections were identified in the United States. Of these, Aspergillus, Pneumocystis, and Candida infections made up roughly 76.0% of the total diagnosed fungal infections (Rayens and Norris, 2022). Nevertheless, the precise role and importance of this colonization remain to be fully elucidated. In addition, it was approximated that around 1.47% of the French population was affected by serious fungal infections annually. Among them, the prevalences of severe asthma with fungal sensitization attacks and allergic bronchopulmonary aspergillosis were rather high (Gangneux et al., 2016).

A number of studies regarding the disease burden of lower respiratory infections worldwide were published (GBD 2015 LRI Collaborators, 2017). However, to our best knowledge, as of now, no comprehensive epidemiological study on lower respiratory tract fungal infections in the global region has been carried out. The understanding and exploration of lower respiratory tract fungal infections is of great significance in the field of medical science and public health. This study utilizes the global data regarding the disease burden caused by lower respiratory tract fungal infections from 1990 to 2021 in the Global Burden of Disease (GBD) Study 2021, aiming to meticulously examine the global distribution of the burden of lower respiratory tract fungal infections, disclose the high prevalence of this disease in geographical regions and age groups, and investigate the correlation between the sociodemographic index and the burden of lower respiratory tract fungal infections.

## Methods

### Data collection

The GBD study is designed to offer comprehensive and up-to-date global, regional, and national data regarding the burden of disease, injury, and risk factors (GBD 2019 Demographics Collaborators, 2020). 12,000 collaborators from more than 160 countries and territories participated in, reviewed, or analyzed readily available data to create GBD 2021 indicators. This study evaluated 459 health outcomes and risk factors throughout 204 countries and territories globally and regionally (GBD 2021 Risk Factors Collaborators, 2024; Murray and GBD 2021 Collaborators, 2024). The estimates for lower respiratory tract fungal infections and associated deaths at GBD database are generated using the Cause of Death Ensemble model (CODEm) methodology, which integrates a range of data sources, including vital registration data, disease registries, health surveys, and expert inputs (GBD 2021 Causes of Death Collaborators, 2024).

We retrieved raw data concerning disability-adjusted life years (DALYs) from the GBD, encompassing estimates of DALYs and deaths in absolute quantities, along with age-standardized rates (GBD 2021 Diseases and Injuries Collaborators, 2024). Regarding the second category, the Socio-demographic Index (SDI), a combined indicator gauging the social and demographic advancement status of countries or regions, classified them into high SDI (> 0.81), high-middle SDI (0.70 - 0.81), medium SDI (0.61 - 0.69), low-middle SDI (0.46 - 0.60), and low SDI (< 0.46) (Bai et al., 2023). SDI values spanning from 1990 to 2021 were acquired from the Institute for Health Metrics and Evaluation (IHME) website (http://ghdx.healthdata.org). Thirdly, the research employed 21 geographic regions based on epidemiological resemblance and geographic closeness, such as Southeast Asia and Andean Latin America. The fourth category consisted of 204 distinct countries or regions (involving 21 countries with subnational locations).

The data of this research were sourced from a publicly accessible database and ethical approval or informed consent was not requisite.

### Disease burden depiction

DALYs indicate the sum of years lost result from premature death (YLLs) and years lived with disability (YLDs), covering the notion of years of healthy life lost. Considering potential fluctuations, Age-standardized DALYs rate (ASDR) and age-standardized mortality rate (ASMR) were computed to accommodate varying age compositions. We employed ASR and average annual percentage changes (AAPC) to appraise trends in DALYs and mortality rates related to this condition. The AAPC, together with its 95% confidence intervals (CI), were ascertained through a linear regression model to measure the changes as time goes on. The population pyramid was utilized to show number and rates changes in age- and sex-specific DALYs and Death. The population was divided into 20 age ranges: under 5 years old, 5 - 9 years old, 10 - 14 years old, 15 - 19 years old, 20 - 24 years old, 25 - 29 years old, 30 - 34 years old, 35 - 39 years old, 40 - 44 years old, 45 - 49 years old, 50 - 54 years old, 55 - 59 years old, 60 - 64 years old, 65 - 69 years old, 70 - 74 years old, 75 - 79 years old, 80 - 84 years old, 85 - 89 years old, 90 - 94 years old, and over 95 years old.

### The relationship between SDI and the burden of ASMR and ASDR in 21 regions and 204 countries

We exhibited the number of deaths due to lower respiratory tract fungal infections and age-standardized mortality rates with 95% uncertainty intervals from 1990 to 2021 in a total of 21 regions and 204 countries and territories globally. As mentioned above, these countries and territories were classified into five SDI regions. It should be noted that a lower SDI used to signify a comparatively low degree of social development.

### Join-point analysis

To present the changing disease load, we adopted join-point regression analysis to scrutinize the time tendencies of ASDR and ASMR of lower respiratory tract fungal infections across different SDI regions. This approach detects notable alterations in trends, named as joint points, and splits the overall trend into distinct sub-segments in accordance with these points. Annual percent change (APC), together with its 95%CI, were computed through log-linear regression to conduct further analysis of the epidemiological trends in each sub-segment.

### Statistical analysis

Variables were presented in the form of numbers, percentages, and ratios. Pearson correlation analyses were performed to evaluate the connections between ASR and SDI in 2021, along with between AAPC and the average SDI through 204 countries and territories. All statistical analyses and visualizations for this research were accomplished using R 4.1.2 software. *P* < 0.05 was adopted to determine statistical significance.

## Results

### Global burden of lower respiratory tract fungal infection from 1990 to 2021

On a global scale, compared with 1990, the absolute burden of lower respiratory tract fungal infection has decreased (**Table 1**). The total DALYs declined from 1315375.93 (95% Uncertainty Interval [UI], 1040494.88 - 1630165.71) in 1990 to 1301752.24 (95% UI, 1113453.22 - 1514947.72) in 2021. Nevertheless, the number of deaths increased from 30359.76 (95% UI, 25326.50 - 35459.58) in 1990 to 45541.64 (95% UI, 39299.35 - 51944.15) in 2021.

**Table 1:** Global mortality and death of lower respiratory tract fungal infections and their AAPC by gender, SDI level, and region.

Characteristics	DALY 1990		DALY 2021		AAPC, % (95% CI)	Mortality 1990		Mortality 2021		AAPC, % (95% CI)
	No. (95% UI)	ASDR (/100,000) (95% UI)	No. (95% UI)	ASDR (/100,000) (95% UI)		No. (95% UI)	ASMR (/100,000) (95% UI)	No. (95% UI)	ASMR (/100,000) (95% UI)	
Global	1315375.93 (1040494.88-1630165.71)	25.86 (20.96-31.29)	1301752.24 (1113453.22-1514947.72)	16.37 (13.89-19.15)	-1.46 (-1.58 to -1.35)	30359.76 (25326.50-35459.58)	0.75 (0.63-0.87)	45541.64 (39299.35-51944.15)	0.56 (0.48-0.64)	-0.91 (-1.05 to -0.77)
Gender										
Male	702434.44 (556571.07 -873194.86)	28.88 (23.64-34.75)	731846.36 (629664.72-848360.63)	19.01 (16.30-22.09)	-1.33 (-1.48 to -1.19)	16256.44 (13771.13-19022.47)	0.91 (0.78-1.06)	24870.73 (21906.32-28112.86)	0.68 (0.60-0.77)	-0.88 (-1.1 to -0.67)
Female	612941.48 (474244.10-767352.67)	23.43 (18.53-28.81)	569905.88 (461443.97-669793.02)	14.04 (11.35-16.63)	-1.64 (-1.77 to -1.51)	14103.32 (11577.46-16811.99)	0.64 (0.52-0.75)	20670.91 (16828.45-24337.52)	0.46 (0.37-0.54)	-1 (-1.22 to -0.78)
SDI rank										
High SDI	79088.71 (70719.51-87097.43)	7.61 (6.80-8.40)	99905.35 (89188.08-109721.26)	5.08 (4.59-5.54)	-1.3 (-1.83 to -0.77)	4,267.98 (3,690.13-4,777.28)	0.40 (0.34-0.45)	6,216.68 (5,294.26-6,973.91)	0.26 (0.23-0.29)	-1.38 (-1.87 to -0.89)
High-middle SDI	109,705.86 (93,047.61-127,435.30)	11.48 (9.70-13.41)	114,467.27 (104,400.90-125,836.58)	6.63 (6.00-7.27)	-1.7 (-2.4 to -1)	3196.33 (2759.84-3634.90)	0.37 (0.32-0.43)	5383.29 (4746.11-6014.26)	0.29 (0.26-0.33)	-0.67 (-1.2 to -0.13)
Middle SDI	348,496.94 (281,913.01-419,948.70)	23.86 (19.67-28.13)	315,973.72 (278,106.16-357,101.07)	12.85 (11.27-14.55)	-1.98 (-2.13 to -1.82)	7,839.25 (6,513.24-9,141.93)	0.79 (0.66-0.93)	12,454.78 (10,795.68-14,077.51)	0.54 (0.46-0.61)	-1.24 (-1.46 to -1.02)
Low-middle SDI	421,313.36 (322,135.74-530,506.82)	38.17 (30.50-46.55)	393,726.19 (330,648.28-470,210.89)	24.73 (20.80-29.43)	-1.4 (-1.54 to -1.27)	8,360.30 (6,706.41-10,130.06)	1.14 (0.93-1.37)	12,405.98 (10,459.11-14,741.87)	0.93 (0.78-1.11)	-0.65 (-0.98 to -0.31)
Low SDI	355804.12 (262196.54-466928.11)	73.00 (56.70-90.52)	376537.53 (290835.58-476443.52)	46.26 (37.19-56.31)	-1.46 (-1.6 to -1.32)	6670.38 (5111.41-8317.45)	2.22 (1.74-2.75)	9039.12 (7280.06-10951.80)	1.67 (1.35-2.01)	-0.91 (-1.06 to -0.76)
GBD regions										
Andean Latin America	15070.24 (11899.02-18300.12)	47.33 (38.99-56.16)	16671.48 (13366.88-20549.71)	27.63 (22.17-34.02)	-1.72 (-2.28 to -1.17)	390.65 (325.08-460.70)	1.78 (1.49-2.11)	711.78 (570.36-868.19)	1.244 (0.99-1.51)	-1.18 (-2.01 to -0.33)
Australasia	741.36 (655.99-821.36)	3.44 (3.04-3.81)	1187.67 (991.27-1352.58)	2.19 (1.87-2.48)	-1.51 (-2.78 to -0.22)	41.96 (36.10-47.28)	0.20 (0.17-0.23)	88.29 (71.08-101.83)	0.14 (0.11-0.16)	-1.19 (-3.53 to 1.21)
Caribbean	6928.21 (5460.25-8465.54)	21.93 (17.60-26.17)	8911.61 (7406.01-10735.92)	17.80 (14.71-21.43)	-0.67 (-0.95 to -0.39)	199.45 (164.86-234.43)	0.79 (0.666-0.93)	364.14 (309.88-426.67)	0.68 (0.58-0.80)	-0.52 (-0.87 to -0.18)
Central Asia	19070.97 (15167.45-23631.56)	24.08 (19.55-29.09)	14645.76 (12378.74-17116.81)	15.72 (13.33-18.30)	-1.36 (-1.61 to -1.1)	298.96 (246.30-354.79)	0.45 (0.38-0.52)	347.80 (298.41-399.05)	0.42 (0.36-0.48)	-0.16 (-0.51 to 0.2)
Central Europe	13995.47 (12459.65-15553.46)	10.87 (9.56-12.17)	16675.61 (15120.61-18257.70)	9.12 (8.29-9.97)	-0.55 (-1.05 to -0.04)	480.39 (431.50-533.39)	0.36 (0.32-0.40)	775.90 (700.94-850.37)	0.36 (0.33-0.40)	0.12 (-0.41 to 0.66)
Central Latin America	29097.95 (23845.45-34439.96)	21.47 (18.26-24.86)	38070.63 (32327.89-43959.16)	15.23 (12.91-17.63)	-0.92 (-1.46 to -0.38)	697.28 (598.04-803.47)	0.77 (0.66-0.89)	1385.11 (1188.63-1605.01)	0.57 (0.49-0.66)	-1 (-1.57 to -0.43)
Central Sub-Saharan Africa	39607.83 (28400.94-53615.76)	90.15 (65.22-122.89)	55924.56 (39965.38-75800.42)	71.86 (50.93-98.39)	-0.73 (-0.83 to -0.63)	822.87 (603.93-1120.31)	3.13 (2.24-4.27)	1528.57 (1081.25-2094.90)	2.88 (2.04-3.96)	-0.27 (-0.4 to -0.14)
East Asia	200734.63 (157819.72-248591.48)	20.90 (16.69-25.39)	78221.19 (65145.40-94965.05)	4.61 (3.85-5.52)	-4.82 (-5.13 to -4.51)	4761.58 (3828.63-5648.60)	0.73 (0.59-0.87)	4248.13 (3486.02-5243.55)	0.25 (0.20-0.31)	-3.51 (-3.83 to -3.18)
Eastern Europe	14303.12 (12728.27-15822.07)	5.99 (5.27-6.69)	24746.40 (21773.30-27884.93)	8.89 (7.83-10.03)	1.52 (0.17 to 2.9)	378.60 (339.13-416.76)	0.15 (0.13-0.16)	765.08 (676.64 -861.62)	0.25 (0.22-0.28)	2.07 (0.47 to 3.7)
Eastern Sub-Saharan Africa	149774.56 (110269.21-198443.76)	92.74 (71.40-115.61)	155972.73 (124363.64-193041.47)	57.64 (46.88-69.37)	-1.51 (-1.63 to -1.4)	2948.84 (2272.75-3713.07)	3.04 (2.36-3.84)	3907.88 (3178.08-4707.84)	2.20 (1.80 -2.63)	-1.04 (-1.13 to -0.94)
High-income Asia Pacific	19847.58 (17673.13-21755.86)	10.83 (9.58-11.92)	23693.75 (19869.05-26857.53)	4.63 (4.05-5.14)	-2.81 (-3.39 to -2.23)	1086.08 (938.54-1213.05)	0.63 (0.54-0.71)	1822.07 (1463.33-2098.29)	0.28 (0.24-0.32)	-2.67 (-3.23 to -2.11)
High-income North America	23008.96 (20575.30-25303.35)	6.79 (6.09-7.44)	27845.14 (25004.52-30725.78)	4.79 (4.33-5.28)	-1 (-1.82 to -0.19)	1231.02 (1058.26-1383.75)	0.34 (0.30-0.39)	1427.19 (1231.89-1594.73)	0.21 (0.19-0.24)	-1.53 (-2.15 to -0.9)
North Africa and Middle East	76027.17 (58100.91-97165.69)	22.96 (18.47-27.84)	63460.73 (53458.69-74902.87)	12.49 (10.61-14.54)	-1.92 (-2.11 to -1.74)	1433.08 (1155.71-1726.94)	0.67 (0.56-0.80)	2012.14 (1700.24-2321.13)	0.49 (0.42-0.57)	-0.99 (-1.22 to -0.76)
Oceania	2527.87 (1846.06-3340.03)	42.94 (32.63-54.73)	4365.56 (3147.08-5736.71)	34.99 (26.33-45.66)	-0.66 (-0.83 to -0.49)	47.40 (35.93-60.53)	1.34 (1.05-1.69)	90.43 (68.55-118.42)	1.11 (0.87-1.47)	-0.63 (-0.75 to -0.5)
South Asia	354279.88 (267270.77-450940.82)	34.66 (27.45-43.09)	304192.94 (253328.54-364117.92)	19.98 (16.66-23.88)	-1.8 (-2 to -1.6)	6983.73 (5525.32-8599.36)	1.02 (0.82-1.26)	10143.28 (8424.13-12255.67)	0.76 (0.63-0.93)	-0.86 (-1.41 to -0.31)
Southeast Asia	116933.96 (92229.53-146047.81)	28.49 (23.02-34.93)	124531.94 (105448.09-143549.97)	19.66 (16.50-22.71)	-1.2 (-1.32 to -1.07)	2464.65 (2012.65-2997.30)	0.86 (0.699-1.06)	4700.84 (3850.11-5406.74)	0.85 (0.68-0.98)	-0.08 (-0.17 to 0.01)
Southern Latin America	6538.09 (5758.82-7352.18)	14.33 (12.64-16.16)	17160.11 (15215.22-19044.34)	20.33 (18.06-22.52)	1.26 (0.42 to 2.12)	276.25 (244.26-312.92)	0.66 (0.58-0.75)	908.69 (786.63-1024.40)	1.01 (0.88-1.14)	1.41 (0.35 to 2.49)
Southern Sub-Saharan Africa	24744.57 (20372.96-29687.15)	58.21 (48.50-69.44)	48034.17 (39885.61-56781.38)	68.98 (57.88-81.52)	0.54 (0.22 to 0.86)	566.03 (472.01-675.14)	1.787 (1.49-2.14)	1324.38 (1116.52-1565.98)	2.25 (1.90-2.65)	0.78 (0.51 to 1.06)
Tropical Latin America	28514.12 (23819.85-33047.09)	24.11 (20.51-27.72)	51071.23 (45369.68-57314.06)	20.42 (18.14-22.92)	-0.43 (-0.99 to 0.13)	762.69 (663.69-873.35)	0.87 (0.75-0.99)	2214.31 (1908.69-2502.17)	0.89 (0.77-1.01)	0.22 (-0.28 to 0.71)
Western Europe	29032.74 (25762.14-32095.23)	5.34 (4.76-5.89)	33573.18 (29337.94-37475.10)	3.48 (3.12-3.84)	-1.28 (-2.19 to -0.36)	1787.51 (1531.40-1999.84)	0.31 (0.27-0.35)	2447.57 (2050.58-2770.39)	0.21 (0.18-0.23)	-1.23 (-2.11 to -0.34)
Western Sub-Saharan Africa	144596.64 (104720.03-192573.29)	74.19 (57.15-93.64)	192795.82 (131413.88-257778.84)	53.26 (39.54-68.34)	-1.05 (-1.14 to -0.95)	2700.75 (2069.31-3426.33)	2.26 (1.82-2.78)	4328.06 (3201.96-5581.03	1.94 (1.52-2.38)	-0.48 (-0.57 to -0.39)

DALYs, disability-adjusted life years; AAPC, average annual percentage changes; SDI, socio-demographic index; ASDR, age-standardized disability-adjusted life year; ASMR, age-standardized mortality rate; NO., Number; UI, uncertainty intervals.

After modifying the effects of age structure, the overall load of lower respiratory tract fungal infections showed a downward trend. The ASDR per 100,000 individuals presented a downward trend (AAPC, -1.46 [95% CI, -1.58 - -1.35], *P* < 0.0001). Likewise, ASMR also demonstrated a decreasing trend (AAPC, -0.91 [95% CI, -1.05 - 0.77], *P* < 0.0001). On the whole, the burden of lower respiratory fungal infections was notably decreased, and a downward trend in mortality was witnessed.

### Regional burden of lower respiratory tract fungal infections from 1990 to 2021

**Figure 1:**
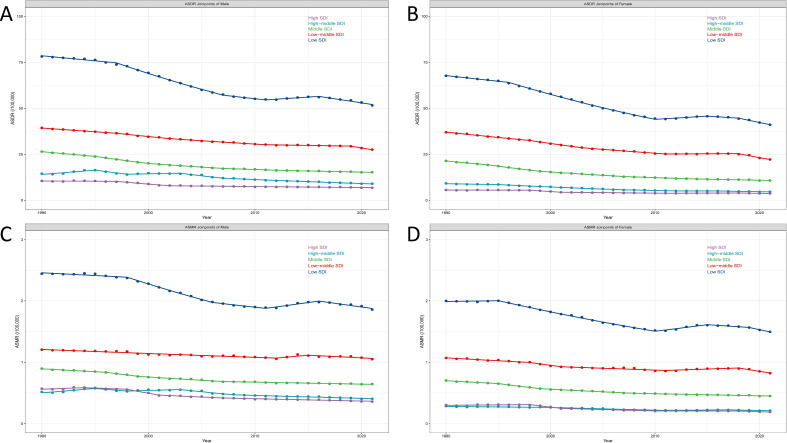
The burdens of lower respiratory tract fungal infection among 204 countries and territories in 2021. **(A)** Regional distribution of the ASDR of lower respiratory tract fungal infection in 2021. **(B)** Regional distribution of trends in ASDR from 1990 to 2021. **(C)** Regional distribution of the ASMR of lower respiratory tract fungal infection in 2021. **(D)** Regional distribution of trends in ASMR from 1990 to 2021. (ASDR, age-standardized disability-adjusted life year; ASMR, age-standardized mortality rate; AAPC, average annual percentage changes; Blue, from light to dark indicates an increase in values; Red: decrease, Purple: increase, the gradient from light to dark represents the intensity of the change).

Regionally, there were significant differences in the burden of lower respiratory tract fungal infections through the 21 geographic super-regions (**Table 1**; **Figures 1A, C**). Sub-Saharan Africa recorded the highest ASDR and ASMR in both 1990 and 2021, although the AAPCs have downward trends. The Eastern Europe experienced notable increases in ASDR from 1990 to 2021 (AAPC, 1.52 [95% CI, 0.17 - 2.9], *P* = 0.028) while East Asia witnessed notable decreases in ASDR during the same period (AAPC, -4.82 [95% CI, -5.13 - -4.51], *P* < 0.0001). Similarly, a remarkable increase in ASMR was observed in Eastern Europe (AAPC, 2.07 [95% CI, 0.47 - 3.7], *P* = 0.011), while a decrease was seen in the East Asia (AAPC, -3.51 [95% CI, -3.83 - -3.18], *P* < 0.0001).

### National burden of lower respiratory tract fungal infections from 1990 to 2021

Nationally, the burden of lower respiratory tract fungal infections presented significant fluctuations between 1990 and 2021. In 2021, the ASDR per 100,000 people ranged from 1.040 [95% UI, 0.66 - 1.45] in San Marino to 126.01 [95% UI, 90.04 - 167.24] in Zimbabwe. Similarly, in 2021, the ASMR ranged from 0.06 [95% UI, 0.041 - 0.089] in San Marino to 4.13 [95% UI, 2.97 - 5.41] in Zimbabwe. During the period from 1990 to 2021, the burden of lower respiratory tract fungal infections shifted considerably in different countries (**Figures 1B, D**). Argentina had the most significant growth in ASDR (AAPC_ASDR_ = 3.02 [95% CI, 2.28 - 3.76], *P* < 0.0001) and ASMR (AAPC_ASMR_ = 3.37 [95% CI, 2.74 - 4.01], *P* < 0.0001), while Finland witnessed the most considerable fall, with AAPCs for both ASDR and ASMR falling beneath -5. Furthermore, China also witnessed a very notable decline, with AAPC_ASDR_ being -5.02 ([95% CI, -5.35 - -4.7], *P* < 0.0001) and the AAPC of the ASMR being -3.69 ([95% CI, -4.64 - -2.72], *P* < 0.0001).

### Burden of lower respiratory tract fungal infections by sex and age group

**Figure 2:**
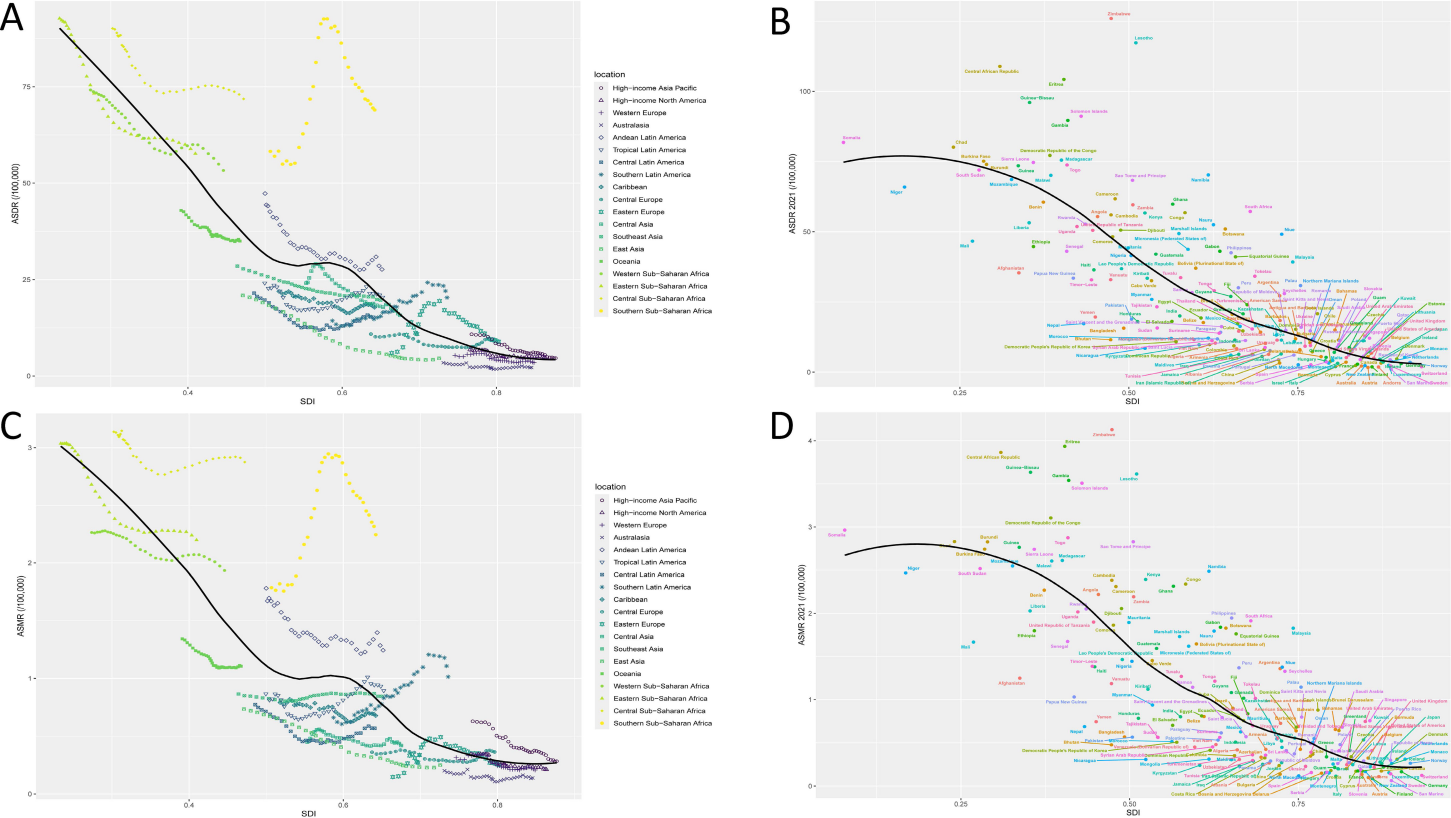
The burdens of lower respiratory tract fungal infection of different age groups from 1990 to 2021. The DALYs **(A)**, ASDR **(B)**, Death **(C)**, Mortality rate **(D)** of different age groups from 1990 to 2021. (ASDR, age-standardized disability-adjusted life year; DALYs, disability-adjusted life years.).

In the sex subgroup analyses, the burden of lower respiratory tract fungal infections was notably higher in males than in females (**Figures 2A, B**; **Supplementary Table 1**). In 2021, the rate of DALY for males was relatively high within the age group of 0-5 years, and then rose with age, reaching a peak at over 95 years old (323.13/100,000 [95% UI, 259.88 - 383.29]). Similarly, in 2021, the rate of DALY for females was relatively high within the age group of 0-5 years, and then rose with age, reaching a peak at over 95 years old (207.32/100,000 [95% UI, 150.69 - 248.70]) (**Figures 2A, B**). In 2021, the number of deaths was relatively high within the age group of 65-69 years for both genders. The highest mortality rates were found in individuals over 95 years old for both genders (**Figures 2C, D**; **Supplementary Table 1**).

### The burden of lower respiratory tract fungal infections according to the SDI

**Figure 3:**
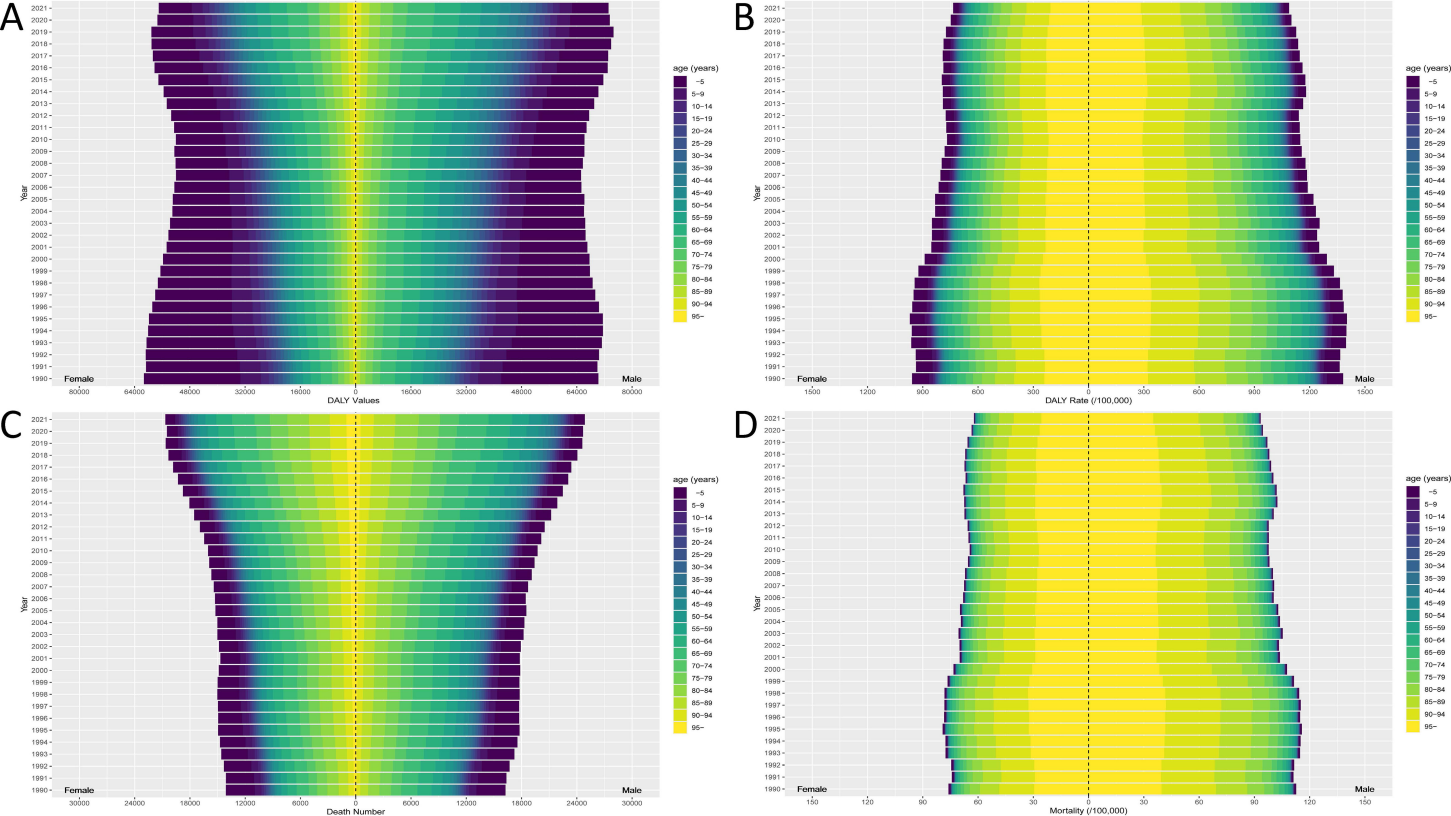
The correlation between SDI and ASR in 19 regions and 204 countries. The correlation between SDI and ASDR in 19 regions **(A)** and 204 countries **(B)**. The correlation between SDI and ASMR in 19 regions **(C)** and 204 countries **(D)**. (ASDR, age-standardized disability-adjusted life year; ASMR, age-standardized mortality rate; SDI, Socio-demographic Index; The black curve represents the fitted curve).

In accordance with the classification of the SDI in 2021, both ASDR and ASMR presented a negative correlation with SDI in the majority of regions. The results suggested that the connections between ASMR/ASDR and SDI differed notably among the 19 geographic regions (**Figures 3A, C**) and 204 countries (**Figures 3B, D**). Countries or regions in the low SDI category displayed the highest ASDR and ASMR.

### The trends of ASDR and ASMR concerning time, sex and SDI

**Figure 4:**
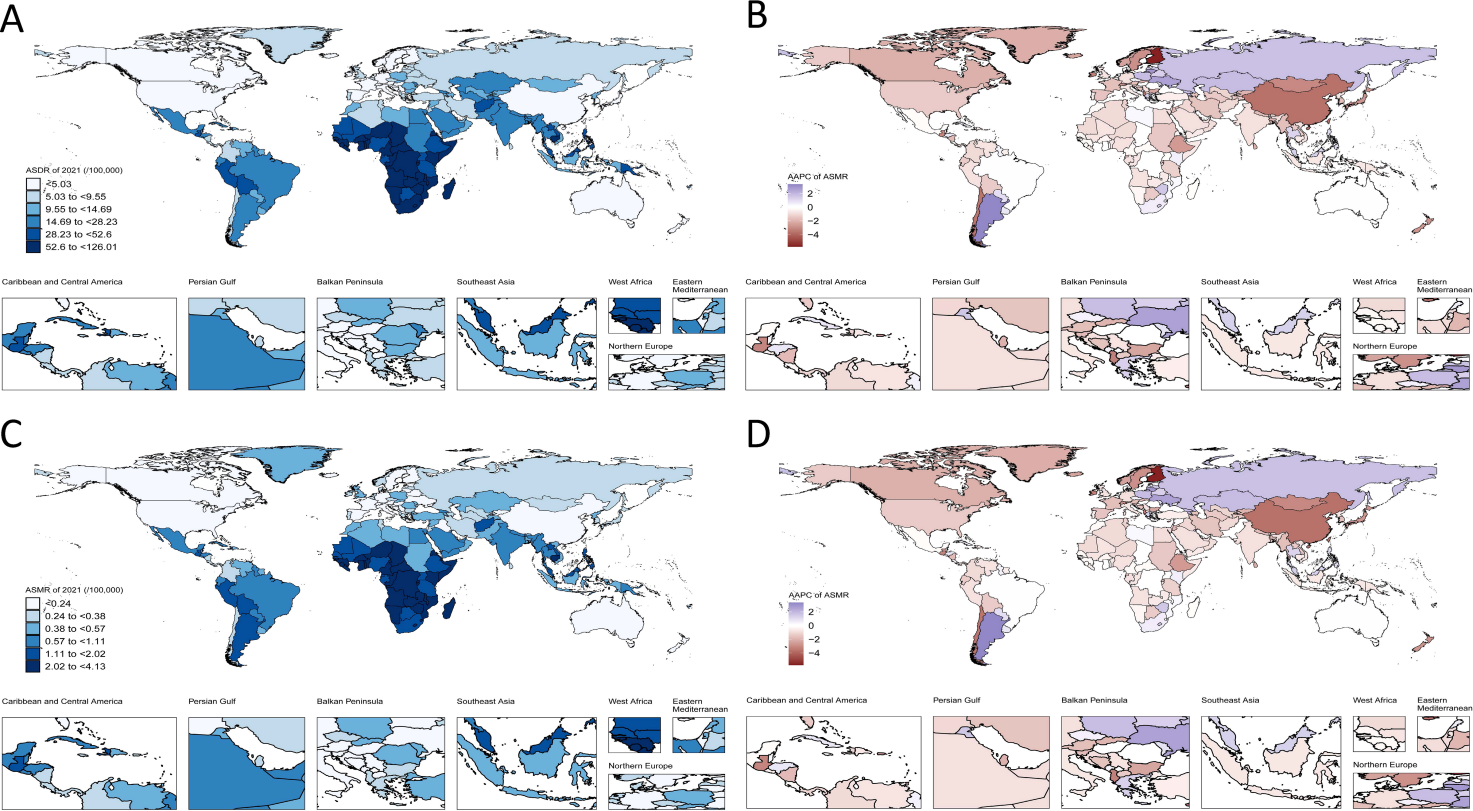
The join-point analysis of ASR change by sex and SDI, 1990 to 2021. The join-point analysis for lower respiratory tract fungal infection in ASDR by SDI of male **(A)**, female **(B)**. The join-point analysis for lower respiratory tract fungal infection in ASMR by SDI of male **(C)**, female **(D)**. (ASDR, age-standardized disability-adjusted life year; ASMR, age-standardized mortality rate; SDI, Socio-demographic Index; Each color represents a different level of SDI, and each point represents a year; For specific join-point years, refer to **Supplementary Table 2**).

Globally, during the period from 1990 to 2021, the ASMR and ASDR of lower respiratory tract fungal infections showed a downward trend. Nevertheless, these temporal trends varied significantly in terms of gender and SDI regions. Among men, the ASDR and ASMR were the highest in the low SDI regions, and the downward trend was the most significant. In contrast, in the high SDI regions, the ASDR and ASMR were the lowest, and the downward trend was the least (**Figures 4A, C**; **Supplementary Table 2**). For women, the trend was alike. The ASMR and ASDR in the low SDI regions were much higher than those in other SDI regions, and the downward trend was the greatest (**Figures 4B, D**; **Supplementary Table 2**).

## Discussions

Lower respiratory infections still constitute a momentous global health issue. A host of studies have utilized the data from the Global Burden of Disease project and undertaken research on the burden of global lower respiratory tract infections from multiple facets (GBD 2021 Lower Respiratory Infections and Antimicrobial Resistance Collaborators, 2024). In 2016, scientists estimated that lower respiratory infections led to 2,377,697 (2,145,584 - 2,512,809) deaths among people of all ages around the world (GBD 2016 Lower Respiratory Infections Collaborators, 2018). Among them, Streptococcus pneumoniae was the main reason for the incidence and fatality of lower respiratory infections on a global scale (GBD 2016 Lower Respiratory Infections Collaborators, 2018). Additionally, the influenza virus is a common cause of acute lower respiratory tract infections worldwide (Li et al., 2024). It has been reported that in 2019, there were around 488.9 million incident cases of lower respiratory infections and 2.4 million deaths (Adesanya and Chiao, 2017). The global age-standardized incidence rate was roughly 6,295 per 100,000 people, and the mortality rate was roughly 34.3 per 100,000 people (Safiri et al., 2023). On a global scale, in 2021, scholars assessed that there were 344 million incident cases of lower respiratory infections, and the estimated quantity of deaths was 2.18 million (GBD 2021 Lower Respiratory Infections and Antimicrobial Resistance Collaborators, 2024). However, up to now, there is no global GBD data for fungal infections of the lower respiratory tract (Li and Nair, 2022).

Globally, common fungi causing lower respiratory infections include species of Aspergillus (Riedling et al., 2024), which are found worldwide. Other filamentous fungi that cause respiratory diseases are Mucorales, black fungi, and species of Fusarium, Scedosporium, and Penicillium. Mucorales are a group of ancient fungi with a global distribution. A recent study found 15 new species of Mucorales, with 12 isolated from South Korea and 3 from Brazil. This indicates that Mucorales have a wide geographic distribution and can be found in both hemispheres (Nguyen et al., 2024). Fusarium species are known for causing crop diseases and are significant mycotoxigenic fungi. They are distributed across various climatic zones worldwide. Climate change is predicted to expand the suitable climate environment for Fusarium spp., potentially increasing their prevalence in agricultural regions. In Europe, the Middle East, and North Africa, Fusarium oxysporum f. spp. is expected to have a wider distribution under future climate scenarios (Ejaz et al., 2023). Penicillium species are commonly found in various environments and are known for their role in food spoilage and as allergens. In Croatia, a study found that Penicillium species were predominant on the surface of traditional meat products, except in the south where Aspergillus species were more prevalent. This indicates that the distribution of Penicillium and Aspergillus species can vary significantly within a single country (Zadravec et al., 2023). The relationship between Candida infection and lower respiratory tract infections has long been a subject of debate. Candida is an important component of the human microbiota. In hospitalized patients, the rate of Candida isolation through active screening is approximately 15%, while in critically ill patients, this rate can reach up to 25% (Epelbaum and Chasan, 2017). Although microbial colonization plays a significant role in secondary infections, there are few reports of Candida pneumonia even in intensive care units (ICU). Therefore, the general consensus is that, in most cases, antifungal treatment for Candida is rarely necessary and should be considered as colonization from respiratory tract samples (Pappas et al., 2016). However, respiratory tract colonization by Candida may serve as an independent risk factor for ventilator-associated pneumonia (VAP), potentially contributing to mucus production, mucus plugging, and atelectasis, which could hold clinical significance (Johnson et al., 2020; Liu et al., 2025).

In accordance with the theory of epidemiological transition, starting from the late 19th century, public health projects and practices have been continuously optimized, and new prevention, diagnosis, and treatment tools for infectious diseases have continued to emerge, resulting in a dramatic decrease in the burden of infectious diseases in the mid-20th century (McKeown, 2009). Consistent with these changes, our research findings reveal that, on a global level, the ASDR and the ASMR showed a downward trend in 2021 compared with 1990. Additionally, the total DALYs went down in 2021, but the number of deaths rose in 2021 when compared with 1990. The AMDR and ASDR of lower respiratory tract fungal infections vary significantly in different countries and regions. There is a significant negative correlation between the SDI and the burden of lower respiratory tract fungal infections.

From the regional point of view, Sub-Saharan Africa recorded the highest ASDR and ASMR in both 1990 and 2021, although the AAPCs have downward trends. The Eastern Europe experienced notable increases in ASDR and ASMR from 1990 to 2021 while East Asia witnessed notable decreases in ASDR and ASMR during the same period. Some studies had indicated that wealth condition (McKeown, 2009), residence location, household dimensions (Akinyemi and Morakinyo, 2018), the kind of toilet facilities, and the sort of cooking fuel were may be associated with acute lower respiratory infections. The highest ASDR and ASMR of lower respiratory tract infections in Sub-Saharan Africa may be related to the relatively poor sanitary conditions and the relatively poor family economic conditions. Over the past few decades, the East Asian region has witnessed high-speed economic growth and development, with its economic strength constantly strengthening. This might be the explanation for the decrease in the ASDR and ASMR of respiratory fungal infections. By contrast, in the Eastern Europe region, the course of economic development has been more circuitous. Many Eastern European countries have experienced the process of economic system transition and reconstruction. Some countries have encountered many challenges in the process of transition. This might be the explanation for the increase in the ASDR and ASMR of respiratory fungal infections.

From the perspective of the country, in 2021, the ASDR and ASMR for lower respiratory tract infections in San Marino were the lowest, while those in Zimbabwe were the highest. San Marino is a tiny country with a small population. Nevertheless, it has a relatively complete medical and health system and a relatively high medical level. In Zimbabwe, adults as well as children were susceptible to respiratory diseases, but the diseases which children and adults were vulnerable to and the causes of death were markedly different (Nyagumbo et al., 2022). One study indicated that roughly 14.9% of Zimbabweans were afflicted with fungal infections every year, and around 80% of them had tinea capitis (Pfavayi et al., 2021). Similar to these research findings, we have also found that Zimbabwe had the highest ASDR and ASMR for lower respiratory tract fungal infection. The possible reasons were as follows. Regarding the health and immune condition of the population, Zimbabwe plagued by issues such as malnutrition, a high prevalence of HIV/AIDS infection, and inadequate management of chronic diseases. These issues had weakened immune function of certain population groups, making them more susceptible to fungal infections. Regarding medical resources, the health system in Zimbabwe was encountering substantial problems (Meldrum, 2008). Its medical and health resources were relatively scarce and it might encounter certain challenges in the diagnosis, treatment, and prevention of diseases. Our research revealed that the ASDR and ASMR in Argentina experienced the most substantial growth. A considerable portion of the residents in Argentina were exposed to the risk of invasive fungal infections, such as immunosuppressed individuals (Gomez et al., 2018), intensive care individuals (Benedetti et al., 2022), or those receiving solid organ and hematopoietic cell transplants (Chaves et al., 2020). Argentina possesses a public healthcare system that is accessible throughout the nation and for everyone, yet it may have restrictions, such as those caused by insufficient funding (Rubinstein et al., 2018). Riera et al. found that in Argentina, no less than 881,023 people suffered from a severe fungal disease every year, and the morbidity and mortality rates were rather high (Riera et al., 2018). The ASDR and ASMR of lower respiratory tract fungal infections in Finland have witnessed the most significant decline (Arvonen et al., 2021). This decline could potentially be attributed to the country’s advanced healthcare infrastructure and public health awareness campaigns.

This positive outcome is the result of a multi-faceted approach adopted by the Finnish authorities and healthcare community. The ASDR and ASMR of lower respiratory tract fungal infections in China have also witnessed a very significant decline. This encouraging development is the outcome of a combination of factors and relevant stakeholders in the healthcare ecosystem. The enhancement of public health awareness and the implementation of preventive measures had a substantial impact. Strengthened infection control practices in healthcare settings, as described in a recent study, have also contributed to reducing the risk of nosocomial infections (Blot et al., 2022). To sum up, the significant decline in the ASDR and ASMR of lower respiratory tract fungal infections in China is the result of comprehensive efforts such as the advancement of medical level, the promotion of public health, and the construction of the health system.

According to the SDI classification in 2021, in most regions, ASDR and ASMR of lower respiratory tract fungal infections were inversely correlated with SDI. Countries in the low SDI category had the highest ASDR and ASMR. In accordance with our findings, Kang et al. indicated that the nations having the lowest SDI suffered from the greatest burden of lower respiratory infections (Kang et al., 2023). This finding is not unexpected and can be attributed to a multitude of factors. In low SDI countries, inadequate sanitation facilities and exposure to environmental pollutants heighten the risk of fungal exposure and infection. Malnutrition is prevalent in these countries, weakening the immune system and rendering individuals more prone to such infections. The impact of polluted air and unhygienic living conditions on facilitating the dissemination of lower respiratory tract fungal infections.

Worldwide from 1990 to 2021, for men and women, the ASDR and ASMR of lower respiratory tract fungal infections were at the peak in low SDI regions, and the downward tendency was the most outstanding. The significant downward trend in low SDI regions indicated that these regions may have taken effective measures to prevent and control the disease, resulting in some improvement. However, in high SDI regions, the ASDR and ASMR were the lowest, and the downward tendency was the slightest. This may indicate that it was difficult to further reduce the infection rate and mortality rate in the region on the already relatively good basis, or more targeted strategies were needed for continuous improvement.

Our research has great significance for clarifying the epidemiology and impact of lower respiratory tract fungal infections, thus aiming to guide healthcare policies. Nevertheless, it should be recognized that this study has some restrictions. Similar to the majority of GBD publications, our research findings rely significantly on mathematical models to assess the disease burden of various nations. This will give rise to errors in GBD data estimation, affecting the quality, accuracy and comparability of our outcomes. In regions with limited medical resources, there may not have sufficient capability to diagnose lower respiratory tract fungal infections. The diagnosis is insufficient and the reporting is deficient, underestimating the burden of lower respiratory tract fungal infections in this population.

## Conclusions

This study carried out a thorough analysis of the burden of lower respiratory tract fungal infections on a global, regional and national scale from 1990 to 2021, uncovering epidemiological trends and regional disparities, which is of vital importance for public health policies and resource allocation.

## Data Availability

Publicly available datasets were analyzed in this study. The original data presented in the study are openly available in data (http://ghdx.healthdata.org/gbd-results-tool).
