# Supplementary material for: Global burden of reported lower respiratory system fungal infection
Source: Front Cell Infect Microbiol. 2025 Feb 14;15:1542922. doi: 10.3389/fcimb.2025.1542922 (PMC11868289; doi:10.3389/fcimb.2025.1542922)
Supplement: Supplementary file 1 [file Table1.docx]

| **Supplementary Table 1. Global mortality and DALYs of lower respiratory tract fungal infections across age in 2021** | | | | |
| --- | --- | --- | --- | --- |
| **Age (years)** | **Male** | | **Female** | |
|  | **Mortality(/100,000) (95% UI)** | **DALYs(/100,000) (95% UI)** | **Mortality(/100,000) (95% UI)** | **DALYs(/100,000) (95% UI)** |
| <5 | 1188.05 (737.78 - 1763.59) | 4285.15 (3300.74 - 5012.22) | 1073.19 (695.92 - 1532.06) | 8164.86 (5934.53 - 9794.56 ) |
| 5-9 | 431.39 (334.60 - 537.27) | 52486.60 (45037.43 - 60764.14) | 383.29 (284.01 - 481.61) | 31596.02 (25901.11 - 37751.04) |
| 10-14 | 245.96 (201.90 - 294.98) | 105499.87 (65535.01 - 156628.67) | 247.41 (188.86 - 304.09) | 95288.69 (61803.51 - 135838.45) |
| 15-19 | 214.84 (179.54 - 254.18) | 35977.00 (27942.85 - 44702.22) | 208.07 (163.41 - 257.49) | 31975.22 (23752.14 - 40119.95) |
| 20-24 | 227.95 (190.26 - 265.65) | 19222.18 (15781.22 - 23021.96) | 211.10 (165.75 - 266.79) | 19306.08 (14780.99 - 23715.60) |
| 25-29 | 283.85 (239.26 - 330.76) | 15714.52 (13122.76 - 18615.45) | 221.09 (173.46 - 267.81) | 15194.46 (11945.75 - 18782.39) |
| 30-34 | 358.38 (301.39 - 415.14) | 15547.23 (12986.66 - 18127.96) | 224.89 (176.98 - 270.30) | 14377.10 (11292.63 - 18134.09) |
| 35-39 | 452.49 (381.24 - 527.31) | 17939.42 (15152.01 - 20899.60) | 265.33 (214.32 - 320.25) | 13971.76 (10979.86 - 16929.71) |
| 40-44 | 584.23 (495.19 - 676.63) | 20844.20 (17537.43 - 24139.41) | 318.19 (254.60 - 386.38) | 13102.57 (10297.07 - 15729.35) |
| 45-49 | 730.01 (619.50 - 849.15) | 24046.65 (20266.31 - 28032.93) | 381.19 (307.56 - 454.22) | 14130.65 (11415.79 - 17040.28) |
| 50-54 | 1362.86 (1168.82 - 1577.01) | 28146.11 (23866.33 - 32596.59) | 819.60 (670.67 - 978.38) | 15362.87 (12306.86 - 18609.66) |
| 55-59 | 1813.04 (1565.45 - 2097.73) | 31605.31 (26804.59 - 36724.46) | 1170.64 (938.46 - 1393.79) | 16543.63 (13354.21 - 19694.84) |
| 60-64 | 2567.29 (2215.50 - 2943.65) | 61183.47 (52802.82 - 70764.21) | 1723.67 (1363.36 - 2033.21) | 39528.40 (31708.48 - 47081.31) |
| 65-69 | 3205.40 (2786.90 - 3695.13) | 74648.21 (64410.91 - 85517.99) | 2439.26 (1991.65 - 2881.65) | 50149.75 (39694.90 - 59121.03) |
| 70-74 | 2065.75 (1797.70 - 2381.74) | 78555.86 (68246.43 - 90508.58) | 1661.60 (1306.63 - 1984.72) | 59737.80 (48818.45 - 70523.71) |
| 75-79 | 2277.76 (1973.89 - 2591.68) | 41648.28 (36283.66 - 48048.98) | 1893.29 (1503.40 - 2254.63) | 33487.48 (26338.41 - 39988.24) |
| 80-84 | 2552.57 (2194.56 - 2908.27) | 36654.29 (31747.66 - 41724.74) | 2349.21 (1855.51 - 2809.06) | 30413.15 (24185.10 - 36215.68) |
| 85-89 | 2349.16 (2006.00 - 2686.64) | 731846.36 (629664.72 - 848360.63) | 2262.76 (1783.10 - 2681.86) | 569905.88 (461443.97 - 669793.02) |
| 90-94 | 1428.76 (1192.98 - 1629.35) | 32089.93 (27628.54 - 36544.76) | 1805.75 (1356.30 - 2131.62) | 29462.39 (23294.26 - 35229.32) |
| 95+ | 531.01 (408.40 - 621.51) | 23389.71 (19974.66 - 26749.87) | 1011.38 (733.48 - 1213.85) | 22504.67 (17783.30 - 26639.73) |
| **Note:** DALYs: disability-adjusted life years; UI: uncertainty intervals. | | | | |

| **Supplementary Table 2. The join-points of ASMR and ASDR by sex in different SDI regions from 1990 to 2021.** | | | | | | | |
| --- | --- | --- | --- | --- | --- | --- | --- |
| **Age** | **Measure** | **Location** | **Years Join-point 1** | **Years Join-point 2** | **Years Join-point 3** | **Years Join-point 4** | **Years Join-point 5** |
|  |  |  | **APC (95%CI)** | **APC (95%CI)** | **APC (95%CI)** | **APC (95%CI)** | **APC (95%CI)** |
| Both | ASMR | Global | 1990-1995 -0.86(-1.45 to -0.27) | 1995-2002*** 0.17(-0.26 to 0.61) | 2002-2010*** -1.85(-2.16 to -1.55) | 2010-2018 -0.95(-1.25 to -0.64) | 2018-2021*** -1.39(-1.63 to -1.16) |
|  |  | High SDI | 1990-1998 -0.6(-0.68 to -0.52) | 1998-2001** -0.7(-1.3 to -0.11) | 2001-2010*** 0.26(-0.24 to 0.77) | 2010-2018 -2.71(-4.93 to -0.43) | 2018-2021* -2.26(-3.63 to -0.87) |
|  |  | High-middle SDI | 1990-1994 -0.56(-0.63 to -0.49) | 1994-2004 -3.06(-4.45 to -1.66) | 2004-2007 1.46(-1.31 to 4.31) | 2007-2021*** -0.2(-0.77 to 0.36) | - |
|  |  | Middle SDI | 1990-1995*** -1.67(-2.98 to -0.33) | 1995-2000*** 2.7(1.42 to 3.99) | 2000-2004* -0.91(-1.27 to -0.55) | 2004-2007* -2.39(-5.4 to 0.72) | 2007-2021*** -2.53(-7.56 to 2.79) |
|  |  | Low-middle SDI | 1990-1997* -0.52(-0.93 to -0.11) | 1997-2000 -1.04(-1.92 to -0.15) | 2000-2011*** -3.98(-8.61 to 0.88) | 2011-2018 0.65(0.18 to 1.12) | 2018-2021** -2.38(-3.52 to -1.22) |
|  |  | Low SDI | 1990-1996 -1.46(-1.75 to -1.17) | 1996-2008*** -0.06(-0.7 to 0.58) | 2008-2011 -1.57(-1.81 to -1.34) | 2011-2016*** -2.9(-5.19 to -0.56) | 2016-2021*** 1.13(-0.43 to 2.71) |
|  | ASDR | Global | 1990-1995*** -0.75(-1.06 to -0.44) | 1995-2007*** -2.19(-2.27 to -2.11) | 2007-2011*** -1.36(-1.93 to -0.78) | 2011-2018* -0.23(-0.43 to -0.04) | 2018-2021*** -2.64(-3.19 to -2.08) |
|  |  | High SDI | 1990-1998 -0.32(-0.87 to 0.23) | 1998-2001** -7.01(-11.35 to -2.46) | 2001-2011*** -1.09(-1.5 to -0.68) | 2011-2018 0.18(-0.55 to 0.92) | 2018-2021 -2.13(-4.35 to 0.13) |
|  |  | High-middle SDI | 1990-1995* 1.61(0.03 to 3.21) | 1995-1998 -4.44(-10.14 to 1.62) | 1998-2003 -0.38(-2.06 to 1.33) | 2003-2007*** -4.77(-6.94 to -2.55) | 2007-2021*** -1.85(-2.04 to -1.66) |
|  |  | Middle SDI | 1990-1995*** -2.4(-2.86 to -1.93) | 1995-2000*** -3.66(-4.25 to -3.06) | 2000-2007*** -2.21(-2.5 to -1.91) | 2007-2013*** -1.36(-1.72 to -1) | 2013-2021*** -0.9(-1.07 to -0.73) |
|  |  | Low-middle SDI | 1990-1997*** -1.32(-1.49 to -1.16) | 1997-2003*** -2(-2.25 to -1.74) | 2003-2011*** -1.37(-1.51 to -1.23) | 2011-2018 -0.09(-0.27 to 0.08) | 2018-2021*** -3.29(-3.79 to -2.78) |
|  |  | Low SDI | 1990-1996*** -0.68(-0.9 to -0.45) | 1996-2008*** -2.6(-2.68 to -2.52) | 2008-2011 -1.19(-2.36 to 0) | 2011-2016** 0.74(0.36 to 1.12) | 2016-2021*** -1.84(-2.1 to -1.57) |
| Male | ASMR | Global | 1990-1995 -1.66(-1.79 to -1.52) | 1995-2007*** -1.47(-1.63 to -1.31) | 2007-2012** -2.14(-2.89 to -1.38) | 2012-2015 0.47(-1.37 to 2.35) | 2015-2021*** 0.25(0.06 to 0.45) |
|  |  | High SDI | 1990-1994 -2.81(-3.38 to -2.22) | 1994-1998 -0.72(-0.83 to -0.62) | 1998-2001* 1.24(0.72 to 1.77) | 2001-2007** 0.13(-0.17 to 0.43) | 2007-2021*** -1.63(-2.1 to -1.17) |
|  |  | High-middle SDI | 1990-1995*** 0.56(0.04 to 1.09) | 1995-1998 -3.31(-4.39 to -2.22) | 1998-2003 -2.03(-3.73 to -0.3) | 2003-2008*** -7.13(-11.35 to -2.71) | 2008-2021*** -2.05(-2.56 to -1.55) |
|  |  | Middle SDI | 1990-1996*** -2.01(-2.11 to -1.91) | 1996-1999* -0.59(-0.8 to -0.37) | 1999-2007*** -0.68(-2.12 to 0.77) | 2007-2021*** 1.21(0.76 to 1.66) | - |
|  |  | Low-middle SDI | 1990-2012*** 0.68(-0.24 to 1.6) | 2012-2015 -1.39(-1.92 to -0.86) | 2015-2021** 1.39(1 to 1.78) | - | - |
|  |  | Low SDI | 1990-1998** -1.89(-1.94 to -1.83) | 1998-2006*** -0.97(-1.18 to -0.77) | 2006-2011** -1.36(-1.67 to -1.05) | 2011-2016*** 1.75(-0.21 to 3.75) | 2016-2021*** -0.45(-0.93 to 0.04) |
|  | ASDR | Global | 1990-1995* -0.48(-0.84 to -0.12) | 1995-2007*** -2.05(-2.15 to -1.95) | 2007-2011** -1.26(-1.9 to -0.6) | 2011-2018** -0.38(-0.6 to -0.16) | 2018-2021*** -2.11(-2.74 to -1.47) |
|  |  | High SDI | 1990-1997 -0.27(-0.96 to 0.42) | 1997-2002*** -5.13(-6.58 to -3.67) | 2002-2021*** -0.75(-0.88 to -0.61) | - | - |
|  |  | High-middle SDI | 1990-1995** 3.09(1.53 to 4.66) | 1995-1998 -4.77(-10.51 to 1.35) | 1998-2003 0.75(-0.99 to 2.53) | 2003-2007*** -4.92(-7.22 to -2.56) | 2007-2021*** -2.12(-2.33 to -1.92) |
|  |  | Middle SDI | 1990-1995*** -2.03(-2.47 to -1.58) | 1995-2000*** -3.45(-4.02 to -2.87) | 2000-2007*** -2.06(-2.35 to -1.78) | 2007-2013*** -1.12(-1.46 to -0.78) | 2013-2021*** -0.78(-0.94 to -0.61) |
|  |  | Low-middle SDI | 1990-1997*** -1.07(-1.26 to -0.87) | 1997-2002*** -1.66(-2.08 to -1.23) | 2002-2011*** -1.15(-1.29 to -1.01) | 2011-2019** -0.31(-0.48 to -0.14) | 2019-2021*** -3.36(-4.6 to -2.1) |
|  |  | Low SDI | 1990-1997*** -0.72(-0.9 to -0.54) | 1997-2007*** -2.65(-2.76 to -2.54) | 2007-2011** -1.15(-1.73 to -0.57) | 2011-2016** 0.69(0.33 to 1.06) | 2016-2021*** -1.59(-1.85 to -1.33) |
| Female | ASMR | Global | 1990-1997*** 0.04(-0.19 to 0.27) | 1997-2001*** -1.73(-2.57 to -0.88) | 2001-2010*** -0.52(-0.73 to -0.3) | 2010-2018* 0.23(-0.26 to 0.72) | 2018-2021*** -2.5(-3.24 to -1.75) |
|  |  | High SDI | 1990-1998* -0.67(-0.95 to -0.4) | 1998-2001** -0.37(-0.59 to -0.15) | 2001-2010*** -6.85(-10.87 to -2.65) | 2010-2018 -0.49(-0.58 to -0.39) | 2018-2021* -1.93(-4.8 to 1.02) |
|  |  | High-middle SDI | 1990-2000** -2.34(-2.58 to -2.1) | 2000-2011*** -2.49(-4.57 to -0.38) | 2011-2017 -1.14(-1.59 to -0.68) | 2017-2021* -1.24(-1.6 to -0.88) | - |
|  |  | Middle SDI | 1990-1995*** 0.92(-0.7 to 2.56) | 1995-1999*** -3.31(-4.74 to -1.85) | 1999-2008*** -1.09(-1.33 to -0.86) | 2008-2021*** -0.05(-0.33 to 0.22) | - |
|  |  | Low-middle SDI | 1990-1998*** -0.78(-1.01 to -0.55) | 1998-2001 -0.8(-1.27 to -0.33) | 2001-2011*** -0.6(-0.98 to -0.22) | 2011-2018** -1(-1.54 to -0.46) | 2018-2021*** -1.82(-2.15 to -1.5) |
|  |  | Low SDI | 1990-1995 -1.06(-1.48 to -0.64) | 1995-2010*** -1.11(-3.48 to 1.31) | 2010-2015*** -6.07(-10.3 to -1.63) | 2015-2019* 0.45(0 to 0.91) | 2019-2021*** -1.75(-2.73 to -0.75) |
|  | ASDR | Global | 1990-1995*** -1.11(-1.43 to -0.79) | 1995-2007*** -2.36(-2.45 to -2.28) | 2007-2010** -1.83(-3.04 to -0.6) | 2010-2018* -0.17(-0.33 to -0.01) | 2018-2021*** -3.26(-3.86 to -2.66) |
|  |  | High SDI | 1990-1998 -0.09(-0.7 to 0.52) | 1998-2001** -7.28(-12.03 to -2.27) | 2001-2011*** -1.34(-1.8 to -0.87) | 2011-2018 0.5(-0.37 to 1.37) | 2018-2021 -2.46(-4.95 to 0.1) |
|  |  | High-middle SDI | 1990-1995 -1.05(-2.33 to 0.26) | 1995-2007*** -3.3(-3.59 to -3.02) | 2007-2012** -2.24(-3.35 to -1.12) | 2012-2016 -0.25(-1.92 to 1.45) | 2016-2021*** -1.98(-2.77 to -1.18) |
|  |  | Middle SDI | 1990-1994*** -2.52(-3.32 to -1.7) | 1994-1999*** -3.97(-4.69 to -3.24) | 1999-2007*** -2.46(-2.73 to -2.18) | 2007-2013*** -1.62(-2.07 to -1.16) | 2013-2021*** -1.03(-1.25 to -0.81) |
|  |  | Low-middle SDI | 1990-1998*** -1.62(-1.81 to -1.44) | 1998-2003*** -2.56(-3.07 to -2.05) | 2003-2011*** -1.57(-1.77 to -1.37) | 2011-2018 0.09(-0.15 to 0.34) | 2018-2021*** -4.4(-5.11 to -3.68) |
|  |  | Low SDI | 1990-1996*** -0.93(-1.12 to -0.75) | 1996-2010*** -2.65(-2.7 to -2.59) | 2010-2015*** 0.78(0.44 to 1.12) | 2015-2018 -0.8(-1.84 to 0.25) | 2018-2021*** -2.69(-3.18 to -2.19) |
| **Note:** SDI: socio-demographic index; ASMR: age-standardized mortality rate; ASDR: age-standardized disability-adjusted life rate; APC: Annual percentage change; * *P* < 0.05, ** *P* < 0.01, *** *P* < 0.001 | | | | | | | |
